# The management of a duodenal fistula involving the right hepatic duct: a rare case report

**DOI:** 10.3389/fmed.2024.1346590

**Published:** 2024-02-01

**Authors:** Qiu Ming, Yang Jun, Luo Nai-wen, Cao Lei, Fan Yu-dong, Wang Shu-guang

**Affiliations:** Department of Abdominal Surgery, Guiqian International General Hospital, Guiyang, China

**Keywords:** choledochoduodenal fistula, the right hepatic duct, bile duct stenosis, bile duct injury, biliary anatomy

## Abstract

The formation of an internal fistula between the biliary system and the gastrointestinal tract is a rare condition with various etiologies, predominantly associated with recurrent chronic inflammation of the biliary system and tumors. Patients with this condition may lack specific clinical manifestations, presenting with symptoms such as abdominal pain, fever, jaundice, or may show no clinical signs at all. Common types of internal fistulas include cholecystoduodenal fistula, cholecystocolonic fistula, and choledochoduodenal fistula. Among these, the right hepaticoduodenal fistula is extremely rare and seldom reported in clinical literature. We herein report a case of right hepaticoduodenal fistula and analyze its mechanism, treatment principles, and preventive measures through a literature review.

## 1 Introduction

The development of an internal fistula linking the biliary system with the gastrointestinal tract is frequently observed in clinical reports, predominantly involving cholecystoduodenal fistula, cholecystocolonic fistula, and choledochoduodenal fistula. This occurrence primarily stems from chronic inflammation triggered by biliary system stones (1) and is also frequently associated with tumors. However, because of the widespread adoption of laparoscopic cholecystectomy, iatrogenic biliary injuries have become the second most prevalent cause of biliary fistulae after cholelithiasis (2, 3). These injuries often result from thermal damage to the biliary system and can be challenging to identify during surgery. The instance of a right hepatoduodenal fistula described in this report is exceedingly rare. By presenting this case and reviewing pertinent literature, readers can gain insight into the mechanisms of occurrence, treatment principles, and preventive measures.

## 2 Case report

**Figure 1:**
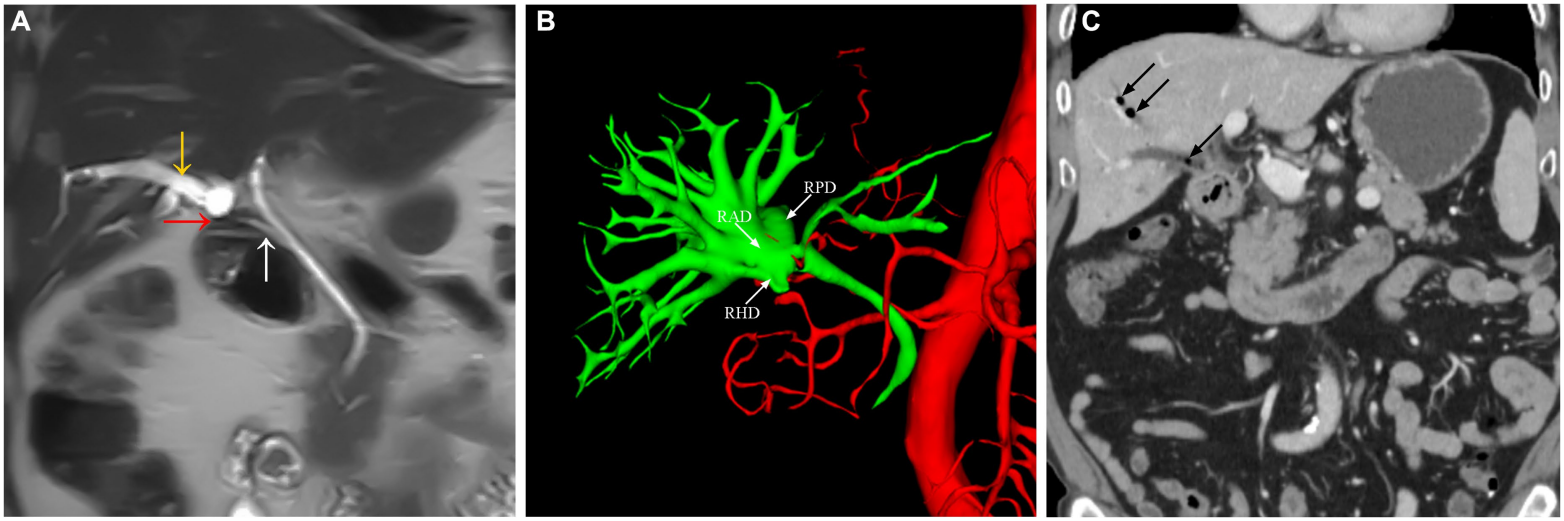
The abdominal MRI scan **(A)** showing the right hepatic bile duct (yellow arrow), a fistula between the right hepatic duct and the duodenal bulb (red arrow), and a low confluence of the right hepatic duct into the common bile duct (white arrow). Three-dimensional cholangiography **(B)** showing the right anterior hepatic duct, right posterior hepatic duct, right hepatic duct, and an abnormal opening with stenosis in the right hepatic duct. The abdominal CT scan **(C)** revealed the presence of gas within the right intrahepatic duct (black arrow).

**Figure 2:**
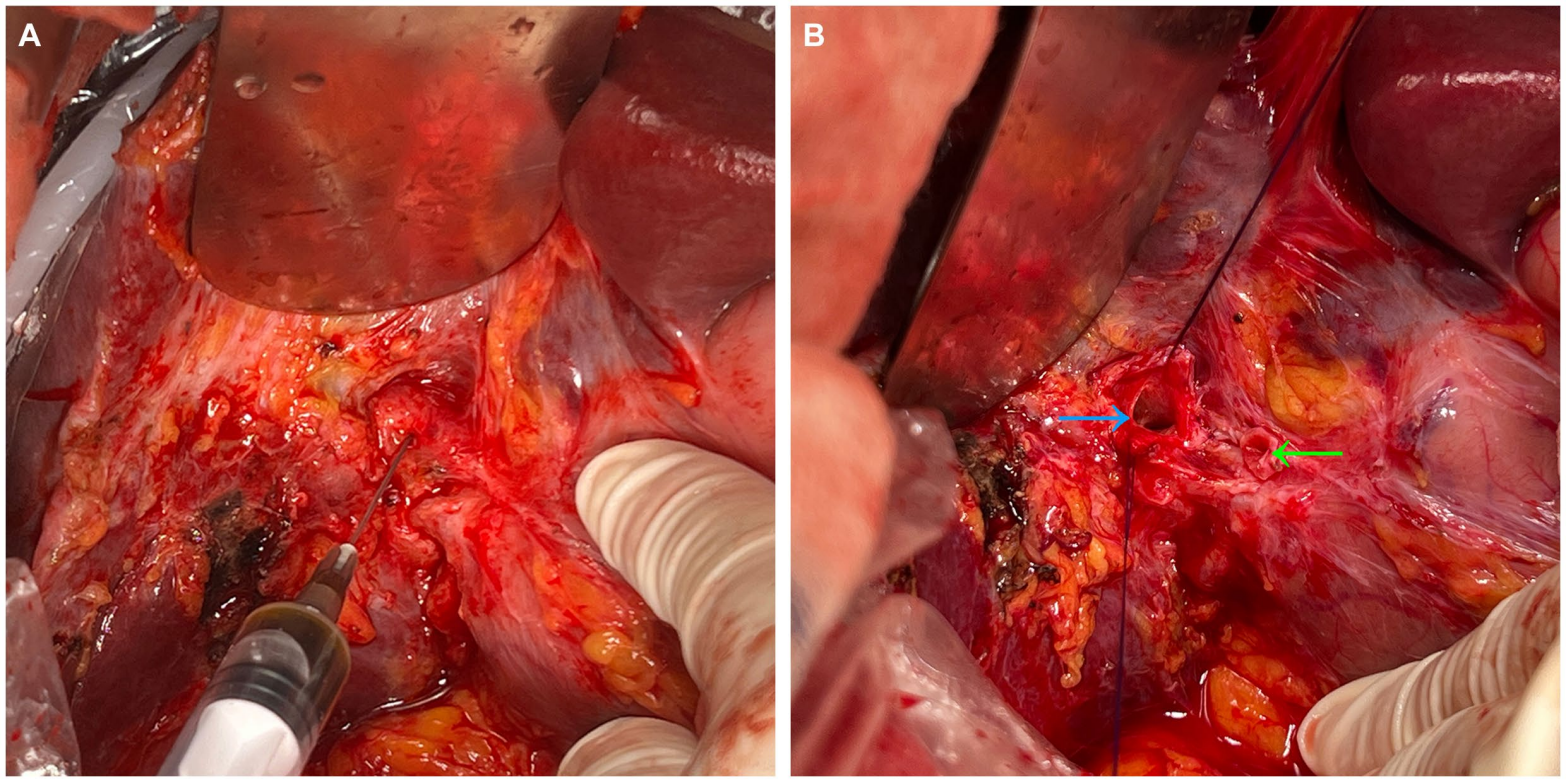
The duodenal bulb adheres to the hepatic hilum **(A)**. A puncture was made in the right hepatic duct, and bile was aspirated. The sinus tract was sharply dissected open **(B)**, which revealed the openings of the right anterior and posterior hepatic ducts (blue arrow), as well as a fistula leading to the duodenum (green arrow).

**Figure 3:**
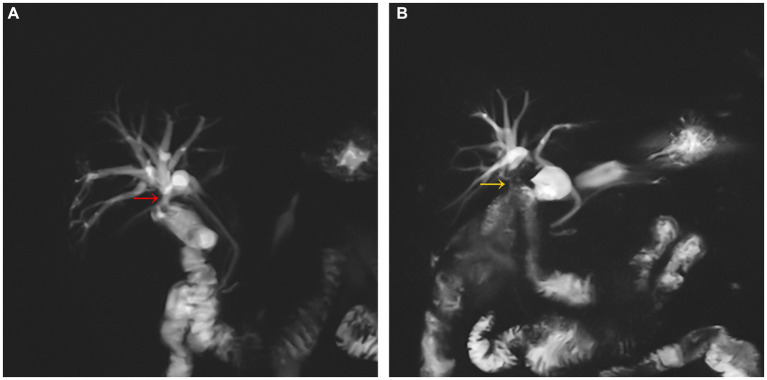
Preoperatively **(A)**, the right hepatic duct communicated with the bulb of the duodenum (red arrow), and the biliary tree of the right side of the liver was dilated. Postoperatively **(B)**, The Rouxen-Y right hepatic duct-jejunum anastomosis (yellow arrow) restore the normal anatomical relationship between the duodenal bulb and the right hepatic duct, and the dilation of the biliary tree in the right side of the liver improved.

A 67-year-old male patient presented with intermittent epigastric pain persisting for a decade. Throughout this period, there were no signs of fever or jaundice. He was admitted to our hospital following an abdominal CT scan indicating right intrahepatic bile duct dilatation and presence of gas within it. Twelve years earlier, he had been diagnosed with gallbladder stones, underwent open cholecystectomy at an external hospital, and was discharged post-surgery without complications. His personal, marital, and family medical history were unremarkable. On physical examination, his temperature was 36.2°C, pulse 76 beats/min, respiration 20 breaths/min, and blood pressure 99/59 mmHg. No jaundice was evident, and cardiopulmonary auscultation revealed no abnormalities. A 10-cm surgical scar was noted in the right epigastrium, with the entire abdomen exhibiting softness, absence of tenderness, rebound pain, muscle tension, or palpable masses. Routine blood tests, liver and kidney function, electrolytes, and tumor markers showed no abnormalities. Upper abdominal imaging [magnetic resonance imaging (MRI) + magnetic resonance cholangiopancreatography (MRCP)] revealed narrowing of the right hepatic duct, suspected choledocholithiasis in the confluence area of the right liver lobe, significant bile duct dilation in the right liver lobe, a low confluence of the right hepatic duct into the common bile duct and close adhesion of the right hepatic duct to the duodenum. Three-dimensional biliary tract reconstruction displayed stenosis at the onset of the right hepatic duct, dilation of the right intrahepatic bile duct, with no anomalies in the left hepatic duct and common bile duct (Figure 1). Based on the patient’s medical history and imaging, the diagnosis was right hepatic duct stenosis and a right hepatic duct-duodenal fistula. An open laparotomy was performed, revealing the right hepatic bile duct adhered to the duodenum, with a firm fibrotic sinusoidal tract. Upon incision and exploration, the sinusoidal tract was found to connect the right bile duct and duodenal lumen, confirming the presence of a right hepatic duct-duodenal internal fistula (Figure 2). Cryopathological analysis of the excised sinusoidal tract tissue indicated inflammatory changes. Cholangioscopic examination of the right intrahepatic tertiary bile duct exhibited notable dilation, chronic inflammatory reactions in the bile duct wall, and flocculent bile sludge, suggesting chronic obstruction of the right hepatic biliary tract and impaired bile drainage. Consequently, the patient underwent resection of the stenotic right hepatic duct, right hepatic duct-jejunum Roux-en-Y anastomosis, and repair of the duodenal fistula. Removal of the gastric tube occurred on the third postoperative day, followed by a transition to a liquid diet. Discharge from the hospital took place 7 days post-operation, and the patient reported no abdominal pain upon discharge. After 1 month post-surgical procedure, a follow-up MRI + MRCP exhibited alleviation of right intrahepatic bile duct dilation (Figure 3). Additionally, the patient’s clinical symptoms of abdominal pain had completely resolved.

## 3 Discussion

The bilioenteric fistula presents with diverse manifestations, sometimes remaining asymptomatic and exhibiting a low incidence in clinical statistics. Predominantly linked to factors such as biliary stones, biliary tract tumors, and iatrogenic injuries, the formation of internal fistulas between the biliary system and the intestine often stems from local chronic inflammation within the biliary system and tumor invasion (4, 5). This specific case involved a patient with a history of cholecystectomy, experiencing recurrent abdominal pain 2 years after the operation, and after excluding biliary tract stones, peptic ulcers, and biliary tract tumors, we hypothesized that iatrogenic insidious injury to the bile duct was the primary cause of the condition. Among iatrogenic causes, thermal injury, particularly in open or laparoscopic cholecystectomy, stands out as the most common (6). Following thermal injury to the bile duct wall, adhesion to adjacent organs occurs, accompanied by local fibrous tissue proliferation. Moreover, the regenerative capacity of the bile duct wall post-thermal injury is limited, rendering self-repair challenging. Consequently, the bile duct wall loses its normal structure, leading to early bile leakage or delayed bile duct stenosis and subsequent biliary endocardial fistulas (7). Clinically, late-onset bile duct stenosis and biliary endocardial fistulas pose challenges for detection due to their insidious nature.

During cholecystectomy, monopolar electrocautery is frequently employed to dissect the gallbladder triangle. While establishing a key safety view helps identify the gallbladder duct, hepatic duct, and common bile duct clearly, instances of biliary thermal injury are rare. However, the presence of anatomical variations significantly increases the risk of such injuries (8, 9). Biliary wall thermal injury incites trauma and inflammation in biliary epithelial cells, stimulating fibroblast proliferation, extracellular matrix secretion, significant collagen deposition, and subsequent bile duct scar tissue formation. This process sees fibroblasts transforming into myofibroblasts, akin to smooth muscle cells, further promoting fibrous contraction in the bile duct tissue, ultimately leading to continuous fibrosis (10, 11). The insidious and clinically challenging nature of late biliary stricture and intrahepatic bile duct injury is evident. Anatomical variations, such as low convergence of the right hepatic duct, further elevate the probability of biliary injury in such cases. Studies reveal that incorrect recognition of local cholecystic anatomy contributes to 76.2% of biliary injuries (12), highlighting factors such as short gallbladder ducts, low convergence of gallbladder ducts, and left and right hepatic ducts. The non-traumatic and non-ionizing nature of MRCP, along with its exceptional ability to provide clear visualization of the biliary system, suggests that it is advisable to perform preoperative MRCP. This facilitates the assessment of abnormal anatomical bile ducts and mitigates the risk associated with iatrogenic bile duct injury (13, 14). Inflammation and intraoperative bleeding also significantly impact the surgeon’s ability to judge surrounding anatomy, increasing the likelihood of bile duct damage when using electrocautery devices (15). To mitigate biliary thermal injury, surgeons should avoid blind use of electrocautery or electrocutting mode separation, minimizing thermal injury in neighboring bile ducts. Practitioners must exercise caution during gallbladder triangle dissection, utilizing electrocautery under a safe view and reducing monopolar thermal penetration frequency near the bile duct while employing less time and current intensity. Anatomical variations stand out as the primary risk factor for iatrogenic bile duct injury, objectively heightening the risk during surgery. Additionally, the chief surgeon’s role cannot be understated in biliary thermal injury. Reviewing data reveals that surgeons undergoing systematic cholecystectomy training can reduce the incidence of biliary injury; however, 97% of errors stem from subjective perception errors (16). This underscores the importance of surgeons at all levels of hepatobiliary surgery avoiding overconfidence during procedures. It is recommended that surgeons minimize monopolar electrocautery use near crucial anatomical structures such as the bile duct, fully comprehending the ramifications of biliary injury. The continuous emphasis on preventing biliary thermal injury remains paramount.

Upon analyzing the patient’s MRCP and three-dimensional reconstruction of the bile duct, we determined that the intrahepatic bile duct was classified as type I (17). However, due to it being the second operation and the absence of imaging data from the first operation, we were unable to classify the extrahepatic bile duct. Nevertheless, an internal fistula had formed between the right hepatic duct and the duodenum. This type of biliary tract fistula is uncommon. The dilation of intrahepatic bile ducts disrupts a stable biliary environment, leading to recurrent biliary inflammation, dysbiosis of the biliary microecology, and the formation of bilirubin stones (18, 19). Additionally, it may result in periductal portal hypertension and induce liver atrophy (20). Certain treatments, such as endoscopic or percutaneous transhepatic biliary dilatation or stenting, may be considered in specific cases, such as bile leakage, biliary peritonitis, or early biliary strictures (21). However, these approaches may result in long-term frequent reflux cholangitis and fail to effectively eradicate the diseased bile duct. Consequently, some experts advocate surgical repair as the preferred treatment for this condition (22). The surgical approach aims to eliminate the diseased bile duct, alleviate biliary obstruction, establish unobstructed bile drainage, prevent complications, and close the duodenal fistula. Ideally, the surgical strategy involves removing the affected bile duct while preserving the physiological biliary tract pathway. However, in cases of an internal fistula, excising the required length of the bile duct is often challenging, making it difficult to perform anastomosis of the bile duct ends. Patients with this condition often require a biliary-enteric anastomosis procedure. In this instance, after considering various treatment options, biliary reconstruction was specifically requested by the patient. So we opted to excise the narrow bile duct of the right hepatic duct and perform a right hepatic duct-jejunum Roux-en-Y anastomosis, coupled with repairing the duodenal fistula. During biliary-enteric anastomosis, attention is crucial to prevent anastomotic stenosis, which correlates closely with the surgeon’s expertise. Specialized hepatobiliary surgeons are recommended for these procedures, significantly improving surgical success rates and reducing complications (23). Additionally, selecting the appropriate bile duct suture material is essential. In our center, we used 5–0 or 6–0 monofilament absorbable sutures (Polydioxanone Suture) for biliary-enteric anastomosis, placing knots outside the anastomotic site to prevent a foreign body reaction caused by nonabsorbable suture materials in the bile duct. Prolonged stimulation can lead to local granulation tissue, increasing the risk of bile duct stenosis. Follow-up results align with Shahin Hajibandeh et al.’s findings (24) regarding the outcomes of different bile duct suturing methods, confirming that proficient suture techniques significantly reduce biliary stricture incidence post-biliary surgery.

## 4 Conclusion

Despite being extremely rare, a right hepatic ductal duodenal fistula was observed in this case. While there is no direct evidence linking this case to cholecystectomy, our analysis, combined with literature review, emphasizes the necessity for biliary surgeons to underscore the importance of biliary tract variations, standardize electrosurgical instrument usage, and rationally select biliary sutures and suture methods. Additionally, attention must be given to the detrimental effect of heat on the biliary tract, emphasizing the sensitivity of biliary epithelial cells to heat and nonabsorbable substances, and reducing occult biliary tract injuries due to nonstandardized operations. The treatment principle involves removing diseased bile ducts, restoring biliary patency, and closing the fistula. Whenever feasible, maintaining the normal physiological bile duct passage is preferred; however, if not viable, bile-intestinal anastomosis remains the best alternative in such cases. The present study solely constitutes a case report, thereby limiting its generalizability. Further investigation is warranted to comprehensively elucidate the pathophysiology and treatment strategies pertaining to this particular disease.

## Data availability statement

The original contributions presented in the study are included in the article/supplementary material, further inquiries can be directed to the corresponding author.

## Ethics statement

Written informed consent was obtained from the individual(s) for the publication of any potentially identifiable images or data included in this article.

## Author contributions

QM: Writing – original draft, Writing – review & editing. YJ: Supervision, Writing – review & editing. LN-w: Writing – review & editing. CL: Writing – review & editing. FY-d: Project administration, Writing – review & editing. WS-g: Project administration, Writing – review & editing.
